# Nanoarchitectonics of Illite-Based Materials: Effect of Metal Oxides Intercalation on the Mechanical Properties

**DOI:** 10.3390/nano12060997

**Published:** 2022-03-18

**Authors:** Jiwei Jia, Daoyong Wu, Yu Ren, Jiyu Lin

**Affiliations:** Key Laboratory of Karst Georesources and Environment, Ministry of Education, College of Resources and Environmental Engineering, Guizhou University, Guiyang 550025, China; j18392199861@163.com (J.J.); 18208475707@126.com (Y.R.); linjy718@163.com (J.L.)

**Keywords:** clay minerals, metal oxides, mechanical properties, molecular dynamics

## Abstract

Clay minerals inevitably interact with colloidal oxides (mainly iron and aluminum oxides) in the evolution of natural geomaterials. However, the interaction between the clay minerals and the colloidal oxides affecting the stability and the strength of geotechnical materials remains poorly understood. In the present work, the interaction between the clay minerals and the colloidal oxides was investigated by reaction molecular dynamics simulations to explore the mechanical properties of illite-based materials. It was found that the metal atoms of the intercalated amorphous iron and aluminum oxides interact with oxygen atoms of the silica tetrahedron at the interface generating chemical bonds to enhance the strength of the illite-based materials considerably. The deformation and failure processes of the hybrid illite-based structures illustrated that the Al–O bonds were more favorable to the mechanical properties’ improvement of the hybrid system compared with Fe–O bonds. Moreover, the anisotropy of illite was greatly improved with metal oxide intercalation. This study provides new insight into the mechanical properties’ improvement of clay-based materials through metal oxides intercalation.

## 1. Introduction

A large number of clay minerals are widely distributed in the natural rocks and soils [1,2,3]. The evolution of clay minerals in geological history inevitably interacts with iron and aluminum oxides [4,5], playing a decisive role in the strength and stability of geotechnical engineering. Many geological disasters, such as landslides [6,7,8], foundation instability [9,10], and drilling collapse [11,12], are closely related to clay minerals. Recently, clay minerals are extensively used in modern industry as a necessary industrial raw material [13,14], and the hydration, adsorption, and swelling along with the thermodynamic properties of clay minerals have become research hotspots in the fields of environment and chemical engineering. Illite/iron nanoparticles were synthesized using a liquid-phase reduction method to remove Pb(II) from an aqueous solution [15]. Pore fluid pressurization caused by the confinement of water in nanometric micropores is one of the main causes of soil instability [16]. Scholars pay more attention to inhibiting clay swelling in the oil industry [17].

Regarding the mechanical properties of clay minerals, many experimental methods, including nanoindentation [18], ultrasonic pulse [19], inelastic neutron scattering [20], and Brillouin scattering [21], were proposed to obtain the strength and deformation moduli. Additionally, computational simulation methods were developed to interpret the experimental results, playing an important role in understanding the structural and dynamic properties of clay minerals. In particular, density functional theory [22,23,24] and molecular dynamics simulation [25,26,27] are widely used to simulate the mechanical properties of clay minerals. For example, with the help of molecular dynamics simulation, the atomistic scale deformation and failure processes of illite [28], kaolinite [29,30,31], and montmorillonite [32,33] are found to be dominated by the number of broken bonds and their corresponding broken sequences. Actually, the experimental and computational results were carried out by pure clay minerals. However, clay minerals inevitably interact with the iron and aluminum oxides in the evolution of rocks and soil. Previous research [34,35] has pointed out that the interaction between colloidal oxides and clay minerals determines the strength of the mixtures. However, the mechanism of interaction between the clay minerals and the colloidal oxides as well as the strength formation and deterioration of clay-based materials remains a persistent fundamental challenge in the geosciences.

In this work, four hybrid structures of illite crystals and amorphous metal oxides (i.e., Al_2_O_3_, Fe_2_O_3_, and FeO(OH)) were constructed according to the X-ray diffraction (XRD) results of natural lateritic soil. Then, the interactions between the illite mineral and the colloidal oxides were investigated by molecular simulation based on the ReaxFF force field, aiming to explore the strength formation and deterioration of clay-based materials at the atomistic scale. The stretching and shearing mechanical performances of the clay-based hybrids were studied in detail, especially the stress–strain relationship, peak strength, and elasticity moduli as well as the deformation and failure processes. Additionally, the formation and fracture of chemical bonds were employed to explain the mechanism of interaction between the clay minerals and the colloidal oxides, which determines the strength formation and deterioration of clay-based materials. This study provides new insight into the improvement in the mechanical properties of clay-based materials by metal oxides intercalation.

## 2. Methods

### 2.1. Models

**Figure 1:**
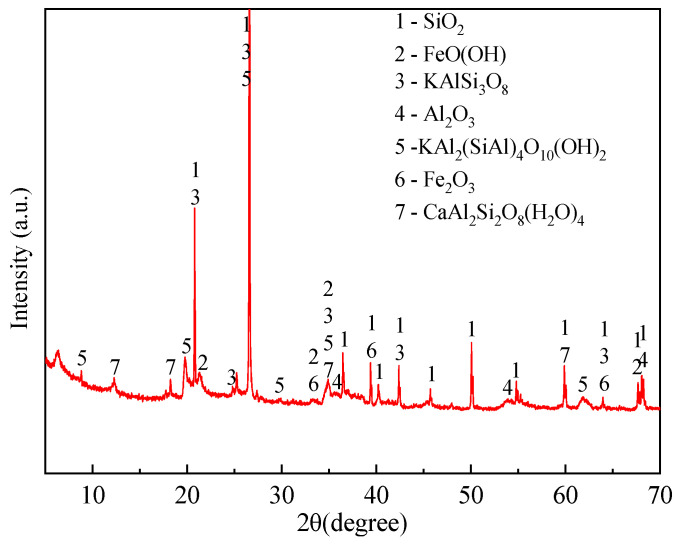
The XRD pattern of lateritic soil.

The first key challenge of our study was to determine the critical chemical components affecting the physical and mechanical properties of natural lateritic soils. The chemical components of this mixture adopted in our study were measured by XRD (shown in Figure 1). The main minerals of the mixture and their weight content can be obtained by the full-peak fitting performed on the XRD pattern (listed in Table 1). The main clay mineral is illite, which the ratio to total clay minerals is 59.5%, and the colloidal oxides are mainly FeO(OH), Al_2_O_3_, and Fe_2_O_3_, the ratio of total colloid to soil was 49.3%. Noteworthily, the crystallinities of colloidal oxides were 39.17% from XRD, indicating that most of the colloidal oxides exist in the amorphous form. Here, the crystal morphology of oxides, quartz, potassium microcline, and anorthite were regarded as weathering residues in the mixture, which are inert constituents compared with the clay minerals and soil colloids. Therefore, the colloidal oxides were regarded as amorphous metal oxides and the influence of inert components was not considered, and in the process of modeling, established to simplify the calculation models.

**Figure 2:**
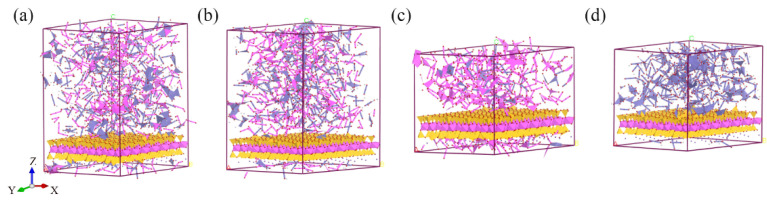
Four hybrid structures of illite-based materials (**a**–**d**), respectively, represent the molecular structure of illite-based models A, B, C, and D.

The hybrid illite-based structures of illite crystals and amorphous metal oxides were established according to the chemical components and weight contents of illite and colloid oxides listed in Table 2. In addition, the different simulation conditions were set-up to determine the influence of each component on the mechanical properties of the illite-based structures (as shown in Figure 2). Firstly, a hybrid illite-based structure (marked as Model A) intercalated three kinds of amorphous metal oxides (i.e., Al_2_O_3_, Fe_2_O_3_, and FeO(OH)) when the illite crystal layers was established. To conform to the results of the XRD test in Table 1, the number of different oxides molecules was confirmed in Table 2 based on 56 illite molecules. Moreover, if put into engineering, getting rid of oxides is far less difficult than other ways. Therefore, two of the three amorphous metal oxides were severally intercalated with illite crystal layers to establish three illite-based structures (marked as B, C, and D) secondly. To establish amorphous metal oxides layers, the method from Wu et al. was adopted [36], the initial temperature of metal oxides was fixed at 6000 K to mix the metal oxides completely and eliminate the effects of the initial distribution. In addition, the four structures were created by surface modules of Materials Studio, which connected the amorphous metal oxides layer to the surface of illite crystals. The corresponding values of the model parameters are listed in Table 2.

### 2.2. MD Simulations

The MD simulations were performed with the 29 September 2021 software version of LAMMPS software [37], and the Jan 22 software version of VESTA [38] was used in this study for visualization. The three-dimensional periodic boundary condition with a timestep of 0.25 fs was applied in all simulations. The reactive force field (ReaxFF) can be utilized to simulate the chemical reaction and mechanical tests. Van Duin [39] had originally developed the ReaxFF force field to make practical the molecular dynamics simulation of large-scale reactive chemical systems for the hydrocarbons. With the development of molecular dynamics simulations, some scholars [40,41,42] adopted the ReaxFF force field to simulate the response process and mechanical properties of clay minerals. The ReaxFF force field can reasonably simulate the fracture and recombination of chemical bonds. The short-range interaction was determined by bond length and order, while a complicated function calculates the long-range Coulombic interaction. This computational strategy avoids the tedious setting of parameters for the bond and nonbonded interactions [43,44]. Therefore, the interaction potential parameters among atoms in the four hybrid structures, except potassium atoms, were based on the ReaxFF force field parameters published by Y. Zheng et al. [45].

The total potential energy, $E_{total}$, for the interactions of atoms in the four hybrid illite-based structures, except for potassium atoms, are expressed as:

(1) $$E_{total}=E_{VDW}+E_{Coul}+E_{bond}+E_{over}+E_{under}+E_{val}+E_{pen}+E_{tors}+E_{conj}$$

where $E_{VDW}$,$\;E_{coul}$, and $E_{bond}$ are van der Waals, Coulombic, and bonded interactions, respectively. $E_{over}$ and $E_{under}$ are under-/over-coordination energy correction terms; $E_{val}$ and $E_{pen}$ are valence angle terms; $E_{tors}$ and $E_{conj}$ are the energy of torsion angle and the contribution of conjugation effects to the molecular energy. The formulas for these energies can be derived from the literature [39].

In previous studies [46,47,48], potassium atoms did not exchange or bond with the other atoms (without chemical reaction). Therefore, the van der Waals potential energy of potassium atoms was extracted by the Lennard–Jones potential. Lennard–Jones potential was adopted to calculate the interaction between potassium atoms (*K*) and the other atoms (*X*):

(2) $$E_{K-X}=4\varepsilon \left[{\left(\frac{\sigma }{r}\right)}^{12}-{\left(\frac{\sigma }{r}\right)}^{6}\right]$$

where ε, σ, and r are the potential well depth, the zero-crossing distance for the potential, and the distance between two atoms.

Figure 1 illustrates the molecular structures information. Atoms in the above four illite-based structures are differently arranged along, and perpendicular to the layers; thus, their mechanical properties are anisotropic. Therefore, by continuously increasing deformation along their corresponding axes, three uniaxial tension tests (along with *X*-, *Y*-, and *Z*-loading directions) were respectively carried out on an equilibration configuration with constant volume and 300 K (canonical ensemble (NVT)). Firstly, the energy minimization was conducted under vacuum at 0 K. After the minimization, the ReaxFF force field was used for molecular dynamic simulations at 1 atmosphere and 300 K (constant-pressure, constant-temperature ensemble (NPT)) for 100 ps. All the simulated experiments were carried out at the strain rate of 1 × 10^8^ s^−1^. The cell angles were changed during deformation. The motion of atoms was integrated using a Verlet leapfrog algorithm with a time step of 0.25 fs, and the Nose–Hoover thermostat and barostat were used in temperature and pressure control, respectively. The atomic positions from the initial state to the accomplished state during the simulation processes were outputted to evaluate the changes in the microstructure of the system. Finally, the interactions between the illite and the colloids were simulated, and corresponding mechanical properties including the stress–strain curves, strength, and elastic moduli were studied.

In MD simulations, the stress was determined by summing the stress tensor of the individual atoms and dividing them by the initial system area, and the strain was determined by a deformation rate of supercell side length.

### 2.3. DFT Simulation

Due to the reactive force field set bonds being flexible, the bond may play unexpectedly. It is necessary to verify the effect of amorphous metal oxides on mechanical properties by density functional theory (DFT). The calculation of mechanical properties at the electronic structure level was performed on models of the unit cell of illite with different amorphous metal oxides using DFT. We employed the local-density approximation (LDA), PAW pseudopotentials, an energy cutoff at 600 eV, and plane waves as implemented. In addition, because of the low content of iron oxides, the illite models with Al_2_O_3_ or FeO(OH) intercalation were simulated the stretching by DFT.

First, 130 steps of geometry optimization of the respective unit cell were performed using the conjugate gradient method. The final energy (convergence 10^−6^ eV) and cell parameters were recorded for the next simulation step. Second, a sequence of strains was applied to the optimized unit cell, represented by a strain tensor. In general, to obtain the mechanical properties, they were varied in the order +0.02, +0.04, …, +0.30, resulting in several sets of 15 strained unit cells in different directions.

## 3. Results of MD Simulation

### 3.1. The RDF Curves

**Figure 3:**
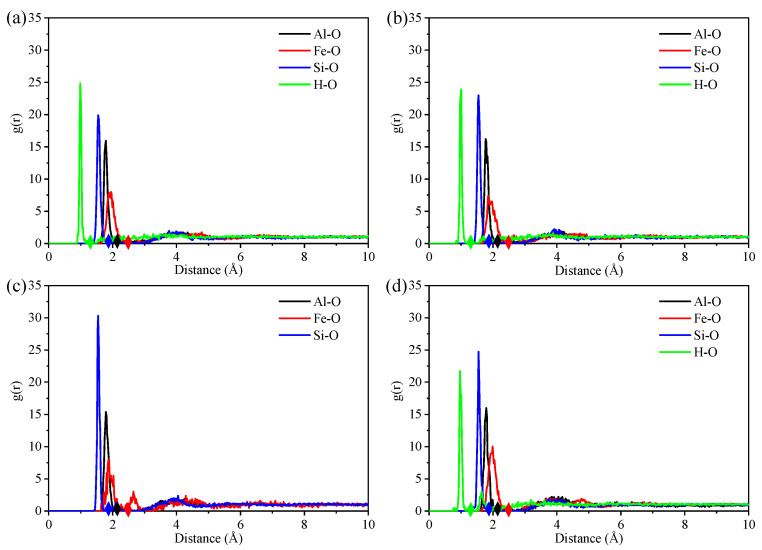
The RDF curves of Al–O, Fe–O, Si–O, and H–O. (**a**–**d**) respectively correspond to A–D structures. Four color rhombuses, respectively, correspond to the first minimum value of each RDF curve.

The variations in mechanical properties during the stretching process were caused by the change in chemical bonds. In this study, the radial distribution functions (RDFs) were employed to set the criteria for determining the fracture of bonds. After optimizing the models, the RDF curves reflected the distribution characteristics and thermodynamic properties of atoms in the illite–metal oxide systems:

(3) $$g_{\alpha \beta }\left(r\right)=\frac{n_{\beta }}{4\pi \rho_{\beta }r^{2}dr}$$

where $\rho_{\beta }$ is the number density of the atoms β; r→r+dr is the distance between atoms α and β; $n_{\beta }$ is the number of atoms β. The RDF curves of Al–O, Fe–O, Si–O, and H–O are plotted in Figure 3. The first minimum value of the RDF curves means that the chances of finding atom pairs with the separation distance were almost zero. The interaction force between two bonded atoms is approximately zero at the first minimum distance. Consequently, we assumed that the bond was broken when the distance between atom pairs was larger than the first minimum value of the RDF curves [29]. Moreover, the hydrogen bond is defined as the distance beyond the H–O bonds and below 2.1 Å. Thus, the first minimum value of the RDF curves of the system is calculated in Table 3. The ratio of chemical bonds of the four hybrid structures is listed in Table 4 to study the effect of the intercalation on the mechanical properties of illite-based structures. As an initial configuration, since several metal oxides exist, the contained metal–oxygen bonds in structure A showed a more balanced state than the other three structures. While due to the decrease of iron and aluminum oxide content (Table 2 shown), the Fe–O and Al–O bond contents decreased respectively in structures C and D (Table 4 shown). Because of the low content of Fe_2_O_3_, the Fe–O bonds of structure B decreased less. The content of the Al–O bonds compared with the composition of aluminum content approached 1.5:1, while the other bonds approached 1:1 compared with the corresponding metal atoms content. Moreover, as Table 2 and Table 4 show, when the structure removed a certain oxide, the total bond content decreased, causing the bond content unrelated (or less relevant) to this certain oxide to increase.

### 3.2. Uniaxial Tension Test

#### 3.2.1. The Tensile Stress–Strain Curves

**Figure 4:**
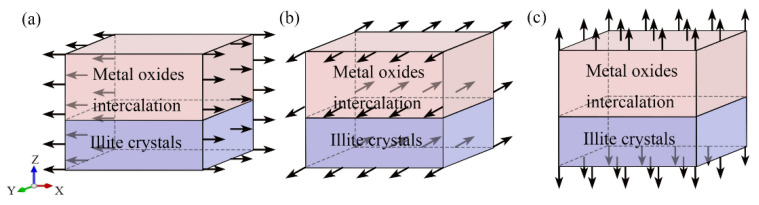
Uniaxial tension along the (**a**) *X*-, (**b**) *Y*-, and (**c**) *Z*-directions. The arrows represent the uniaxial tension direction.

**Figure 5:**
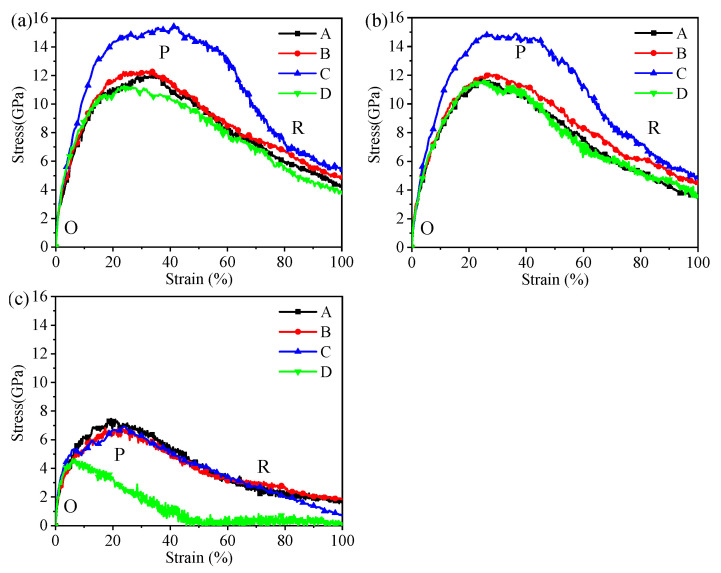
The tensile stress–strain curves along the (**a**) *X*-, (**b**) *Y*-, and (**c**) *Z*-directions. O, P, and R, respectively, represent the original state, the peak stress state, and the residual strength state.

The hybrid illite-based structures were tensile along the *X*-, *Y*-, and *Z*-directions (Figure 4), and the corresponding tensile stress–strain curves are shown in Figure 5. The typical stress–strain curves experienced several stages of linear increase, non-linear increase, and then decreased to the residual value with increasing strain. These stages, respectively, correspond to the elastic deformation, plastic hardening, and failure of the hybrid system. Interestingly, the stress–strain curves in the *X*- and *Y*- directions displayed the same tendency but showed a significant difference in the z-direction. The mechanical properties of the hybrid illite-based structures in the *X*- and *Z*-directions will be discussed in detail because of the similarity in the performances in the *X*- and *Y*-directions.

To further analyze the influence of different amorphous metal oxides on the mechanical properties of the hybrid illite-based structures, the tensile strength and Young’s moduli were calculated according to the tensile stress–strain curves. The Young’s moduli, *Y_α_*, can be estimated by the elastic stage of illite-based structures:

(4) $$Y_{\alpha }=\frac{\sigma_{\alpha \alpha }}{\varepsilon_{\alpha \alpha }}$$

where $\sigma_{\alpha \alpha }$ and $\varepsilon_{\alpha \alpha }$ are, respectively, the stress and strain in the α-direction.

**Figure 6:**
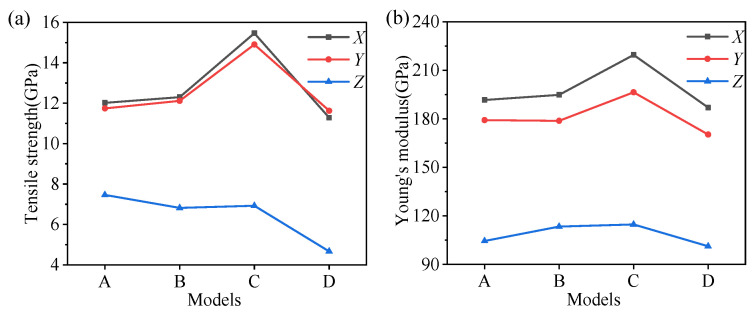
(**a**) The tensile strength and (**b**) Young’s moduli of four hybrid structures in *X*-, *Y*-, and *Z*-directions.

The calculated tensile strength and the Young’s moduli are summarized in Figure 6. The minimum tensile strength was obtained in the *Z*-direction for each hybrid structure, showing very strong anisotropy. The tensile strength of structure D was only 4.67 GPa in the *Z*-direction, which fluctuated around 7 GPa for the other structures. There were few differences in the tensile strength along the *X*- and *Y*-directions. In addition, the tensile strength of structure C was approximately 4 Gpa higher than that of the other structures in the *X*- and *Y*-directions. The variation in the Young’s moduli was similar to the tensile strength along the *X*- and *Y*-directions. However, the amorphous metal oxides had little impact on the Young’s moduli in the *Z*-direction, implying the comparable energy barriers for the deformation of the hybrid structures.

#### 3.2.2. Deformation and Failure Processes of the Hybrid Structures during Tensile

**Figure 7:**
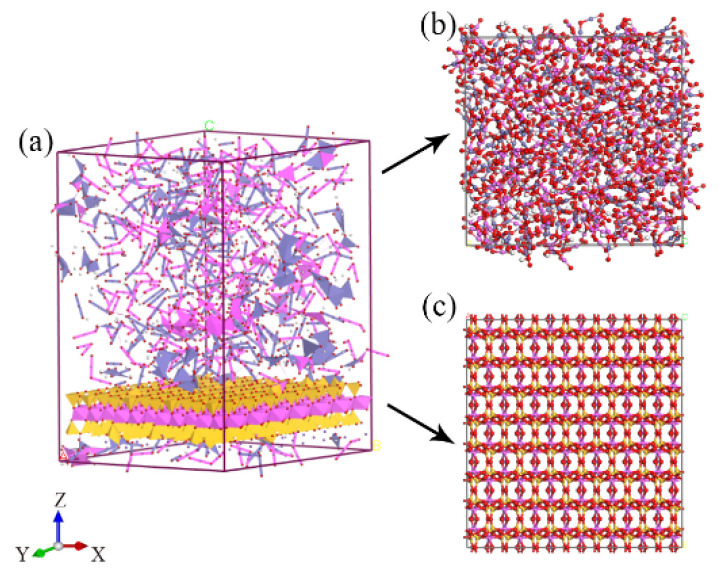
The models of (**a**) structure A, (**b**) amorphous metal oxide intercalation, and (**c**) illite crystals.

For the *X*- and *Y*-directions, the reason for the similarity of the stress–strain curves can attribute to the microstructure of the hybrid structures. Actually, the hybrid illite-based structures were composed of amorphous metal oxides and illite layers (as shown in Figure 7a). The amorphous metal oxides intercalation exhibited isotropic mechanical properties due to the random arrangement of metal oxides (Figure 6b). While illite belongs to T–O–T layered clay minerals, consisting of aluminum chains sandwiched between two silicon chains, which shows the similarity of the microstructure along the *X*- and *Y*-directions (Figure 6c). Therefore, the overall hybrid illite-based structures exhibited similar mechanical properties in the *X*- and *Y*-directions.

**Figure 8:**
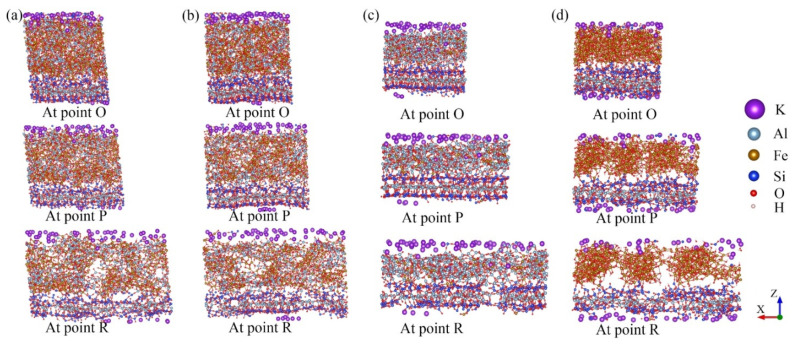
The three-stage models of illite-based structures along the *x*-direction. (**a**–**d**) Represent, respectively, the molecular structure of models A, B, C, and D.

As shown in Figure 5a, the stress–strain variation tendencies of the hybrid structures were roughly the same. The stress required for the structural failure was the largest when the hybrid illite-based structure did not contain FeO(OH) (structure C). In addition, the peak and residual stresses of structure C were much higher than the other structures. The stretching processes of the structures along the *X*-directions are shown in Figure 8. Obviously, the main difference in the microstructure between structure C and the other structures lies in the type and content of chemical bonds in the amorphous metal oxide intercalation. The chemical bonding types of structures A, B, and D amorphous metal oxide intercalation mainly included Fe–O, O–H, OH…O, or Al–O bonds, which were Fe–O and Al–O bonds for structure C. Moreover, the amount of Fe–O bonds in the metal oxide intercalation of structure C was minimal. As mentioned previously, chemical bonds were the fundamental cause of the change in the tensile stress–strain curves. The main reason for the difference in the tensile stress–strain curves was the changes in the bonds. The normalized changes in the bonds during stretch along the *x*-direction are listed in Table 4, where the negative value means new bonds were formed.

Figure 8 exhibits that due to the crack propagation and failure of the hybrid illite-based structures, the number of broken bonds increased gradually during the stretching process (as shown in Table 5). The sequences of broken bonds resulted from both the bond strength and the number of bonds for each type in the *x*-direction. The largest percentage of hydrogen bonds (OH…O) was broken at the peak and residual states in the *x*-direction. The percentage of broken Fe–O bonds was larger than that of the Al–O bonds, and the smallest percentage of Si–O bonds was broken during tension. It can be concluded that the influence of H–O bonds on the mechanical properties of the hybrid structures was negligible due to the lower percentage of bonds in the system and the bare variation of them. By comparing the bond-breaking ratios and the tensile stress–strain curves of structures A and B, it was obvious that the strength of the Al–O bonds was higher than that of the Fe–O bonds. Namely, the Al–O bonds were beneficial to the mechanical properties of the hybrid system. It should be pointed out that the Si–O bonds belonged to the illite crystal plate. The largest percentage of Si–O bonds was broken in structure C. This phenomenon resulted in the destruction of the illite crystal plate (Figure 8c). However, the amorphous metal oxide intercalation in structure C was stabilized at the residual state. Consequently, the peak and the residual stresses of structure C were much higher than other structures because of the abundant Al–O bonds in the amorphous metal oxide intercalation. On the contrary, the microstructure of models A, B, and D were destroyed first within the amorphous metal oxide intercalation (Figure 8). The reason was that the strength of the amorphous metal oxides intercalation was lower than that of the illite crystal plates when there include abundant Fe–O bonds; thus, the strength of the illite crystal plates was not destructed ahead of point P. This means the illite crystal plates determined the strength of these hybrid structures in the horizontal directions (*x*- and *y*-directions) if the amorphous metal oxide layer included many Fe–O bonds (namely, structures A, B, and D). Therefore, the stress–strain curves of A, B, and D share the same tendency.

**Figure 9:**
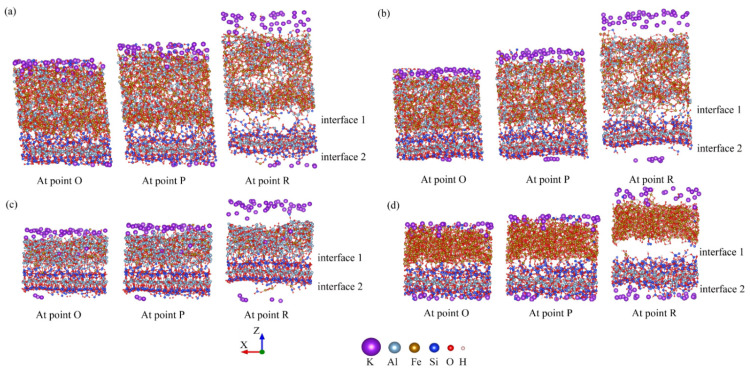
The three-stage models of the structures along the *Z*-direction (**a**–**d**) are, respectively, represented as the molecular structure of models A, B, C, and D.

The mechanical properties of the hybrid structures in the *z*-direction were explored. The variations in the microstructures and the percentage of broken bonds during stretching the four hybrid structures along the *z*-direction are displayed in Figure 9 and Table 5. Interfaces were formed when the amorphous metal oxide intercalation was intercalated in the interlayer of the illite crystal. An interesting phenomenon was observed, where a large number of chemical bonds were generated between the amorphous metal oxide intercalation and the original illite plate at interface 1 (Figure 9). Many Al–O and Fe–O bonds were observed at interface 1 of structures A, B, and C, while much fewer Fe–O and OH…O bonds were detected for structure D. Few of the newly generated chemical bonds were discovered at interface 2, because of the separation layer of the potassium atoms.

In the process of stretching along the *z*-direction, the OH…O bonds were destroyed first due to the fact of their low strength property. Table 3 and Table 6 illustrate that the differences in the total number of chemical bonds and the percentage of broken bonds for structures A and B are ignorable, resulting in the same performances during stretching. The broken Al–O and Fe–O bonds at interface 1 and within the amorphous metal oxide intercalation of structures A and B lead to deformation and failure (Figure 9a,b). Additionally, interface 2 appears at a larger gap compared with interface 1, because the presence of the potassium atoms obstructs the formation of chemical bonds. Notably, new Si–O bonds were generated during stretching by employing the ReaxFF (Table 6). The reason for this phenomenon was that the previously destroyed Si–O bonds of the illite plate caused the combination of silicon atoms with oxygen atoms in the amorphous metal oxide intercalation (Figure 9). More Al–O and Fe–O bonds were generated in structure C at point P. Consequently, the deformation of structure C was mainly located in interface 2, and the deformations of the amorphous metal oxide intercalation r and interface 1 were negligible due to the high strength of the Al–O bonds. Different from that of structures A, B, and C, few Fe–O and OH…O bonds were detected at interface 1 of structure D. Therefore, interfaces 1 and 2 were the weak zones for the hybrid structure D, which disconnected the amorphous metal oxide intercalation from the illite plates during stretching (Figure 9d). As a consequence, the minimum strength was observed in structure D (Figure 9a).

The percentage of broken bonds along the *Z*-direction was much less than that of the *X*- and *Y*-directions, and the interfaces between the illite plate and the amorphous metal oxide intercalation were the weak zones when the hybrid structures were stretched in the *z*-direction, showing very strong anisotropy. Low load-bearing capacity because of the small bond content contributes to the weakest mechanical properties along the *z*-direction compared with that of the *x*- and *y*-directions.

### 3.3. Shear Test

**Figure 10:**
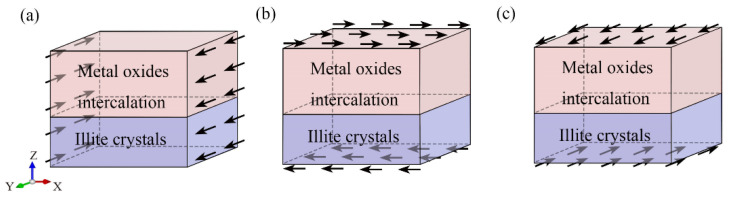
Shear test along the (**a**) *XY*-; (**b**) *ZX*-; (**c**) *ZY*-directions. The arrows represent the shear direction.

**Figure 11:**
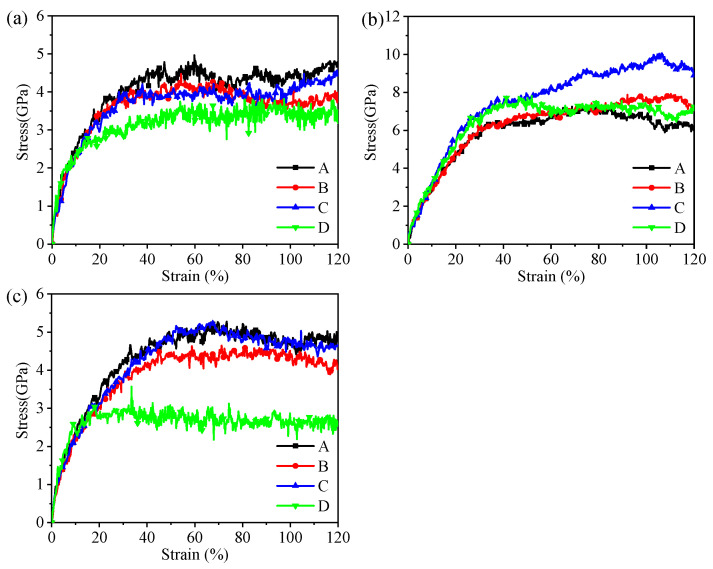
Shear stress–strain curves along the (**a**) *XY*-; (**b**) *ZX*-; (**c**) *ZY*-directions.

The hybrid illite-based structures were sheared along *XY*-, *ZX*-, and *ZY*-directions (Figure 10), and the corresponding shear stress–strain curves are shown in Figure 11. The typical stress–strain curves experience several stages of linear increase, non-linear increase, and then kept a constant value with increasing strain. These stages, respectively, corresponded to the elastic deformation, plastic hardening, and failure of the hybrid system. Stresses in the *XY*-direction were approximately two times higher than those in either the *ZX*- or *ZY*-directions, implying more resistance to in-plane shear rather than transverse shear.

Analogously, the shear strength and shear moduli were calculated according to the shear stress–strain curves. The shear moduli, G, of the structure can be derived from the following equation [49]:

(5) $$G=\frac{1}{V_{0}}{\left(\frac{\partial^{2}E_{str}}{\partial \gamma_{\alpha \beta }^{2}}\right)}_{\gamma_{\alpha \beta }=0}$$

where $V_{0}$ is the volume of the structure; $E_{str}$ is the strain energy; $\gamma_{\alpha \beta }$ is the shear strain in the αβ direction. Equation (5) means the shear moduli, G, is the slope of the stress–strain curve, where $\gamma_{\alpha \beta }=0$.

**Figure 12:**
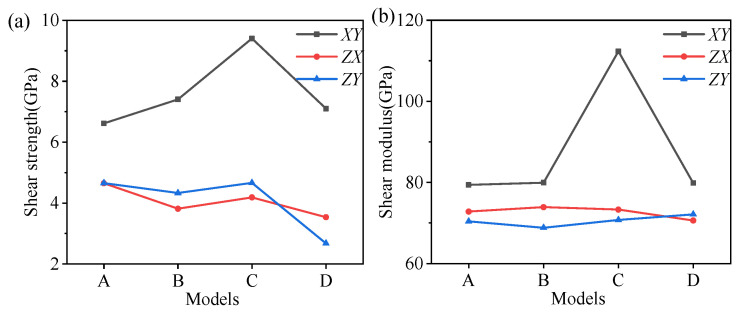
(**a**) The shear strength and (**b**) shear moduli of four hybrid structures.

The calculated shear strength and moduli are summarized in Figure 12. There were few differences in the shear strength along the *ZX*- and *ZY*-directions, and the minimum shear strength was obtained in the *ZX*- or *ZY*-directions for each hybrid structure. The shear strengths of structure D in the *ZX*- or *ZY*-directions were smaller than the other structures (Figure 11a). In addition, the shear strength of structure C was approximately 2.5 Gpa higher than that of the other structures in the *XY*-direction. Compared with the shearing of pure illite conducted by Hantal [28], the intercalation of metal oxides can significantly enhance the shear strength of the hybrid illite-based structures (as shown in Table 7). In the *ZY*-direction, the shear strengths of structures A and D were higher than that of pure illite. The shear moduli of pure illite in the *XY*-, *ZX*-, and *ZY*-directions were 90.1, 9.8, and 8.9, exhibiting very obvious anisotropy. However, little differences in the shear moduli along the *XY*-, *ZX*-, and *ZY*-directions were observed for the hybrid structures. Namely, the isotropic property of illite minerals will be greatly improved by the intercalation of the amorphous metal oxides.

## 4. Results of DFT Calculation and Discussion

Clay minerals inevitably interact with the colloidal oxides (mainly iron and aluminum oxides) in the evolution of rocks and soil. The interaction between the clay minerals and the colloidal oxides plays a decisive role in the stability and strength of geotechnical materials [34,35].

For the clay minerals, the pristine material in this study, the research results of DFT, different force fields, and experiments were shown in Table 8. Moreover, Zartman et al. [50] studied the ClayFF-based method and found corrections for a systematic overestimate of the in-plane stiffness were necessary in comparison to experimental data and electronic structure results. While this enhancement is not much for the layered direction of the layered clay structures (as results in the *z*-direction shown in Table 8). Therefore, it is feasible to improve the mechanical property of lamellar direction of clay by metal oxide intercalation. In the horizontal direction, Hantal et al. said the stress value was considerably higher with ClayFF than the highest value reached with ReaxFF [28], bringing the results based on ReaxFF closer to that based on DFT and experiments, which are also shown in Table 9 and Table 10 in this study. However, after reaching the peak strength, ReaxFF showed a slow decline, while ClayFF did not. The reason for this is that the ReaxFF relies on many fitting functions that cause the bond proximity flexibility, while that based on ClayFF shows rigidity. For the stage after reaching the peak strength, the bond fracture processes gather the focus of this research, which avoids the differences among several methods.

At present, we still know little about the interaction between the clay minerals and the colloidal oxides as well as the strength formation of natural geotechnical materials. Fortunately, the mechanical properties of several pure clay minerals, including illite [28], pyrophyllite [50], and montmorillonite [50], were studied based on DFT. It is well known that both clay minerals and calcium silicate hydrate (C–S–H) are composed of silicon tetrahedrons. It can be seen from Table 8 and Table 9 that the strength of the hybrid structures in the *X*- and *Y*-directions was relatively close to these clay minerals and C–S–H, whatever the force field employed. Notably, the hybrid structural strength in the *Z*-direction was closer to C–S–H rather than the clay minerals. This phenomenon may be attributed to the formation of chemical bonds in the interlayers of the hybrid structures making the microstructures of them similar to that of the C–S–H. Regarding the elasticity moduli of the hybrid structures in the *X*- and *Y*-directions, the calculated values in this study were approximately equal to that of montmorillonite and illite, because the hybrid structures were constructed based on the fundamental T–O–T-layered clay minerals. Additionally, the formation of the chemical bonds in the interlayers of the hybrid structures led to the considerable enhancement elasticity moduli of them in the *Z*-direction compared with the pure clay materials. To further compare the effects of three metal oxides on the hybrid structures, the mechanical properties of three amorphous metal oxides were obtained in the same controlled conditions as the hybrid structures (shown in Table 9). Since amorphous metal oxides are isotropic, we showed the mechanical properties in one direction. As mentioned, aluminum oxides enhance the strength of hybrid structures more than iron oxides, which is consistent with the strength and elastic modulus of aluminum oxide, higher than that of iron oxide. In addition, the strength of amorphous FeO(OH) is much lower than that of the other two oxides, consistent with the highest strength of structure C. However, since pure amorphous metal oxides cannot exist in laterite soil, the feasibility of purifying aluminum oxides is far lower than that for removing iron oxides. Similarly, fewer differences in elasticity moduli between the parallel and perpendicular directions were observed for our hybrid structures, implying that the intercalation of metal oxides reduces the anisotropy of the mechanical properties.

**Figure 13:**
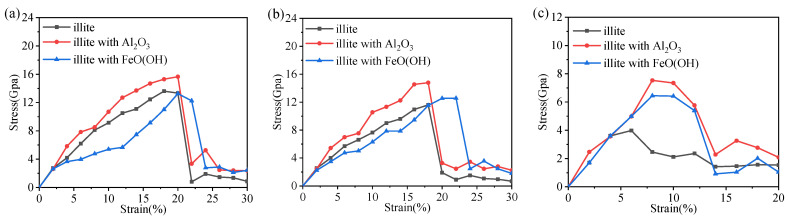
Tensile stress–strain curves in the (**a**) *X*-; (**b**) *Y-*; (**c**) *Z*-directions by DFT calculation.

The simulation results by DFT of hybrid structures are shown in Figure 13 (the stress–strain curves) and Table 9 and Table 10 (tensile strength). We found that for strength values, there was good agreement with the ReaxFF simulation of illite with metal oxide intercalation. However, as much research has shown [53,54,55], the values obtained by DFT simulations were generally smaller than that obtained by ReaxFF simulations, while there is the opposite situation of illite in this study. Notably, this increase can be acceptable, as Zartman et al. found [50], such as mica, the enhancement of the results in strength by DFT simulation is caused by the presence of interlayer cations. Therefore, the ReaxFF simulation is feasible for this study in terms of mechanical properties.

The above discussions demonstrate that the interaction between the clay minerals and the colloidal oxides plays a decisive role in the mechanical properties’ enhancement. But it should be pointed out that the interactions between the clay minerals and metal oxides are extremely complicated in nature. The metal oxides intercalating in the interlayer of clay minerals and coating on the crystal plates may coexist in natural rocks and soils. Large-scale molecular dynamics simulation of smectite clay nanoparticles was carried out by Thomas R et al. [56], and the evolution of microstructural, thermodynamic, and transport properties of a clay nanoparticle suspension during dehydration was calculated and compared to the experiment, providing new insight into the coupled chemistry, mechanics, and transport properties of disordered nanoparticle assemblages. However, the mechanical properties prediction of these media remains a persistent fundamental challenge in geosciences. The simulation results from our study shed light upon the important role of metal oxides in controlling the mechanical properties of clay-based materials.

## 5. Conclusions

The interaction between the clay minerals and the colloidal oxides was investigated by molecular simulation based on the ReaxFF force field to explore the enhanced method on the mechanical properties of illite-based composite materials. Four hybrid structures of illite crystals and amorphous metal oxides (i.e., Al_2_O_3_, Fe_2_O_3_, and FeO(OH)) were established according to the XRD results of natural lateritic soil. After energy minimization, the ReaxFF force field was used for molecular dynamic simulations at 1 atmosphere and 300 K (NPT), and the RDF was employed to determine the formation and fracture of chemical bonds. Then, the mechanical properties of the illite-based hybrid structures, including stretching and shearing in different directions, were studied in detail. The simulation results demonstrated that metal oxides intercalating in the interlayer of illite crystal can significantly improve the mechanical properties of pure illite. Compared with pure illite, the tensile strength increased in the parallel and perpendicular directions when the hybrid structure was abundant in Al_2_O_3_. However, this comparison was based on the ReaxFF force field, and the mechanical property data may have an overestimate than the DFT and experiment methods and below that obtained by ClayFF. The stress–strain curves of the hybrid structures displayed the same tendency in the parallel directions but show a considerable difference in the perpendicular direction, exhibiting the anisotropy of the mechanical properties because the hybrid structures are constructed based on the fundamental T–O–T-layered clay minerals. Additionally, the deformation and failure processes of the hybrid structures were discussed carefully. It was found that the metal atoms of the intercalated amorphous Fe and Al oxides interacted with oxygen atoms of the silica tetrahedron at the interface generating new chemical bonds to enhance the strength of the illite-based composite materials considerably. The microstructure of hybrids abundant in iron oxide were destroyed first within the amorphous metal oxide intercalation but in the illite crystal plate for the aluminum-rich hybrid. Namely, the Al–O bonds were beneficial to the mechanical properties of the hybrid system, because the strength of the Al–O bonds was higher than that of the Fe–O bonds. Furthermore, the anisotropy of illite was greatly improved after metal oxides intercalation. Finally, a brief discussion was carried out considering the mechanical properties of clay-related materials. The simulation results in this study are in good agreement with those obtained by the other scholars. This study focuses on the interaction between the clay minerals and the colloidal oxides providing new insight into the mechanical properties’ improvement of clay-based materials by metal oxides intercalation.

## Figures and Tables

**Table 1:** The chemical components of lateritic soil.

Minerals	Chemical Formula	Content (wt%)	Crystal Cell Volume (Å^3^)
Illite	KAl_2_(SiAl)_4_O_10_(OH)_2_	22.0	940.1
Quartz	SiO_2_	13.8	113.2
Goethite	FeO(OH)	27.0	134.8
Potassium microcline	K(AlSi_3_O_8_)	13.4	735.9
Aluminum oxide	Al_2_O_3_	19.8	235.6
Hematite	Fe_2_O_3_	2.9	97.5
Anorthite	CaAl_2_Si_2_	1.2	1041.5

**Table 2:** Illite-based model parameters.

	Illite-Based Models	A	B	C	D
Compositions	The molecular number of Al_2_O_3_	199	199	199	0
	The molecular number of FeO(OH)	323	323	0	323
	The molecular number of Fe_2_O_3_	27	0	27	27
	The molecular number of illite	56	56	56	56
Cell parameters	x(Å)	34.94	35.57	35.13	34.64
	y(Å)	33.76	33.84	34.99	33.02
	z(Å)	40.32	38.92	26.01	29.87
	v(Å^3^)	47,557.43	46,861.31	31,966.75	34,167.48

**Table 3:** The cutoff distance of individual bond types.

Bond Type	Cutoff Distance (Å)
Al–O	2.1
Fe–O	2.4
Si–O	1.9
H–O	1.2
OH…O	2.1

**Table 4:** The percentage of bonds at each illite-based structure.

Models	Percentage of Bonds (%)				
	Al–O	Fe–O	Si–O	OH…O	H–O
A	40.06	34.98	14.52	3.95	6.49
B	43.70	29.64	16.31	3.44	6.92
C	67.95	5.66	26.39	-	-
D	12.12	53.57	19.15	7.00	8.15

**Table 5:** The percentage of broken bonds along the *x*-direction.

	Models	Percentage of Broken Bonds (%)				
		Al–O	Fe–O	Si–O	OH…O	H–O
At point P	A	4.54	6.50	3.70	27.23	−0.96
	B	6.96	7.16	5.50	12.74	0.63
	C	7.54	7.06	13.87	-	-
	D	8.85	5.46	6.30	24.14	−1.32
At point R	A	5.62	10.51	4.84	27.75	0.00
	B	8.77	9.75	3.09	21.66	−0.63
	C	9.21	14.12	7.19	-	-
	D	14.38	5.66	4.48	22.22	−2.30

The statistics reference the original state of the corresponding bonds, and the negative values represent the formation of the bonds.

**Table 6:** The percentage of broken bonds along the *Z*-direction.

	Models	Percentage of Broken Bonds (%)				
		Al–O	Fe–O	Si–O	OH…O	H–O
At point P	A	1.75	4.31	−1.28	24.61	−0.96
	B	2.61	2.36	−2.95	19.11	0.95
	C	−0.49	−4.71	0.88	-	-
	D	1.11	0.60	−0.56	5.36	−2.30
At point R	A	3.15	6.85	−6.40	20.42	1.27
	B	4.76	5.47	−4.16	19.11	0.32
	C	0.54	−4.71	0.13	-	-
	D	0.66	1.40	−3.78	9.58	−1.32

The statistics reference the original state of the corresponding bonds, and the negative value represents the formation of the bonds.

**Table 7:** Shear moduli of hybrid structures.

		Hybrid Structures				Clay Mineral
		A	B	C	D	Pure Illite [28]
Shear strength (GPa)	*XY*	6.6	7.4	9.4	7.1	-
	*ZX*	4.6	3.8	4.2	3.5	-
	*ZY*	4.7	4.3	4.7	2.7	1.8
Shear moduli (GPa)	*XY*	79.4	79.9	112.3	79.8	90.1
	*ZX*	72.8	73.9	73.3	70.6	9.8
	*ZY*	70.4	68.8	70.8	72.1	8.9

**Table 8:** Mechanical properties of clay minerals by different methods.

		Methods				
		ClayFF [28]	ReaxFF [28]	DFT [29]	DFT [50]	Exp [32]
Tensile strength (GPa)	*X*	-	-	9.5	10	-
	*Y*	10.5 ± 0.5	-	-	-	-
	*Z*	2.3 ± 0.2	2.2 ± 0.1	3.0	-	-
Young’s moduli (GPa)	*X*	225.3 ± 0.7	146.0 ± 44	-	191.5 ± 1.1	176.5 ± 1.1
	*Y*	215.4 ± 0.2	110.0 ± 51	-	181.9 ± 1.5	179.5 ± 1.3
	*Z*	46.9 ± 0.2	24.0 ± 16	-	78.6 ± 3.0	60.9 ± 0.6

**Table 9:** Mechanical properties of several clay minerals and C–S–H in the literature.

		Pyrophyllite [50]	Montmorillonite [50]	Illite [28]	Illite [28]	C–S–H [51]	C–S–H [52]
Tensile strength (GPa)	*X*	-	-	-	-	14.0 ± 0.5	15.0 ± 0.2
	*Y*	10	-	10.5 ± 0.5	-	-	12.5 ± 0.5
	*Z*	3	-	2.3 ± 0.2	2.2 ± 0.1	8.0 ± 0.5	8.0 ± 0.5
Young’s moduli (GPa)	*X*	198.7 ± 0.5	160	225.3 ± 0.7	146.0 ± 44	-	78.0 ± 2
	*Y*	186.0 ± 0.3	160	215.4 ± 0.2	110.0 ± 51	-	118.0 ± 2
	*Z*	70.1 ± 4.8	60	46.9 ± 0.2	24.0 ± 16	-	32.0 ± 2
Force field		DFT	DFT	ClayFF	ReaxFF	ClayFF	ReaxFF

**Table 10:** Mechanical properties of hybrid structures.

		Hybrid Structures				The Oxides in Hybrid Structures			Illite	Illite with Al_2_O_3_	Illite with FeO(OH)
		A	B	C	D	Al_2_O_3_	Fe_2_O_3_	FeO(OH)			
Tensile strength	*X*	12.0	12.3	15.5	11.3	18.9	17.6	14.6	13.6	15.7	13.3
	*Y*	11.7	12.1	14.9	11.6	-	-	-	12.0	14.8	12.5
	*Z*	7.4	6.82	6.9	4.7	-	-	-	3.9	7.5	6.4
Young’s moduli	*X*	191.7	194.9	219.6	187.0	228.0	206.9	205.5	-	-	-
	*Y*	179.2	178.8	196.4	170.3	-	-	-	-	-	-
	*Z*	104.5	113.4	114.7	101.2	-	-	-	-	-	-
Methods		ReaxFF							DFT		

## Data Availability

Some or all data, models, or code generated or used during the study are available from the corresponding author by request (i.e., raw results).
